# Mitochondrial Dysfunction as a Therapeutic Target in Neurodegenerative and Psychiatric Disorders

**DOI:** 10.1192/j.eurpsy.2026.11622

**Published:** 2026-07-31

**Authors:** M. Arts, M. Zeilstra, K. M. Zeilstra-Kalt, L. D. Jonge

**Affiliations:** 1 Geriatric Psychiatry and Neuropsychiatry; 2 Lifestyle Medicine, GGZ WNB, Halsteren; 3 Integrative Medicine, Center de Wetering, Leeuwarden; 4 Integrative Medicine, Leonardo Scientific Research Institute, Groningen, Netherlands

## Abstract

**Introduction:**

Mitochondria are central to cellular bioenergetics, signaling, and apoptosis. Dysfunction in these organelles is increasingly recognized as a common pathway in neurodegenerative and psychiatric disorders. Aberrant mitochondrial dynamics, impaired oxidative phosphorylation, calcium dyshomeostasis, and oxidative stress contribute to neuronal vulnerability, synaptic failure, and altered brain connectivity. Understanding these mechanisms opens novel therapeutic avenues beyond symptomatic treatment.

**Objectives:**

This review synthesizes current evidence on (a) mitochondrial pathophysiology in neurodegenerative and psychiatric disorders, and (b) emerging therapeutic strategies including dietary, pharmacological, and transplantation-based interventions.

**Methods:**

We conducted a literature review of preclinical and clinical studies integrating molecular biology, neuroscience, and psychiatry. Focus on mitochondrial proteostasis, mtDNA alterations, redox balance, and interventional strategies.

**Results:**

*Neurodegenerative disorders* (Alzheimer’s, Parkinson’s, ALS, Huntington’s): mitochondrial dysfunction is linked to impaired energy metabolism, abnormal protein aggregation, and defective mitophagy. In AD, amyloid-β and tau disrupt mitochondrial dynamics; in PD, α-synuclein aggregates impair complex I and trigger ROS.

*Psychiatric disorders* (depression, anxiety, stress-related syndromes): altered mitochondrial morphology, redox imbalance, and disrupted tryptophan metabolism contribute to mood dysregulation. Mitochondrial transplantation normalizes ROS, enhances BDNF signaling, and reduces depressive-like behaviors in models.

*Therapeutic approaches:*

- Dietary interventions: ketogenic diet, carnitine, CoQ10, riboflavin improve mitochondrial biogenesis and antioxidant defense.
- Pharmacological agents: antioxidants (MitoQ, SS-31), AMPK and sirtuin activators, PPAR agonists, and modulators of fission/fusion (Mdivi-1) show preclinical efficacy.
- Mitochondrial transplantation: transfer of viable mitochondria restores neuronal energy supply and improves motor and cognitive function in preclinical models of AD, PD, and depression; early clinical feasibility has been demonstrated in ischemia-reperfusion injury.

**Conclusions:**

Mitochondrial dysfunction represents a convergent mechanism linking neurodegenerative and psychiatric disorders. Advances in mitochondria-targeted therapies, including nutritional strategies, small molecules and organelle transplantation, highlight the potential of moving beyond symptomatic relief toward disease modification. Translation to clinical practice requires standardized biomarkers, optimization of delivery methods, and integration into personalized medicine frameworks.

**Disclosure of Interest:**

None Declared

